# Rapid *Treponema pallidum* Clearance from Blood and Ulcer Samples following Single Dose Benzathine Penicillin Treatment of Early Syphilis

**DOI:** 10.1371/journal.pntd.0003492

**Published:** 2015-02-20

**Authors:** Craig Tipple, Rachael Jones, Myra McClure, Graham Taylor

**Affiliations:** 1 Faculty of Medicine, Imperial College London, London, United Kingdom; 2 West London Centre for Sexual Health, Chelsea and Westminster Hospital NHS Foundation Trust, London, United Kingdom; University of California San Diego School of Medicine, UNITED STATES

## Abstract

Currently, the efficacy of syphilis treatment is measured with anti-lipid antibody tests. These can take months to indicate cure and, as a result, syphilis treatment trials require long periods of follow-up. The causative organism, *Treponema pallidum* (*T. pallidum*), is detectable in the infectious lesions of early syphilis using DNA amplification. Bacteraemia can likewise be identified, typically in more active disease. We hypothesise that bacterial clearance from blood and ulcers will predict early the standard serology-measured treatment response and have developed a qPCR assay that could monitor this clearance directly in patients with infectious syphilis. Patients with early syphilis were given an intramuscular dose of benzathine penicillin. To investigate the appropriate sampling timeframe samples of blood and ulcer exudate were collected intensively for *T. pallidum* DNA (*tpp047* gene) and RNA (*16S rRNA*) quantification. Sampling ended when two consecutive PCRs were negative. Four males were recruited. The mean peak level of *T. pallidum* DNA was 1626 copies/ml whole blood and the mean clearance half-life was 5.7 hours (std. dev. 0.53). The mean peak of 16S rRNA was 8879 copies/ml whole blood with a clearance half-life of 3.9 hours (std. dev. 0.84). From an ulcer, pre-treatment, 67,400 *T. pallidum* DNA copies and 7.08x107 16S rRNA copies were detected per absorbance strip and the clearance half-lives were 3.2 and 4.1 hours, respectively. Overall, *T. pallidum* nucleic acids were not detected in any sample collected more than 56 hours (range 20–56) after treatment. All patients achieved serologic cure. In patients with active early syphilis, measuring *T. pallidum* levels in blood and ulcer exudate may be a useful measure of treatment success in therapeutic trials. These laboratory findings need confirmation on a larger scale and in patients receiving different therapies.

## Author Summary

Syphilis is an infection that is spread both sexually and from mother-to-child. Worldwide, it affects an estimated 11 million people each year. Treatment is available, but relies heavily on penicillin and may not be as effective where the infection involves the brain or nervous system. Clinical trials are needed to assess new treatments, but proving that people are cured with current tests can take months. We developed a new test to measure the success of treatment for the early stage of syphilis. We then used it in four patients and found that the syphilis bacteria were cleared from the blood and ulcer samples collected by 56 hours. All four patients were followed up with normal tests and found to have been cured. Our new test is the first to show the speed of bacterial clearance after treatment and we plan to use it in clinical trials of new treatments. Our data may also mean that patients with syphilis are unable to pass on the infection three days after treatment, but this also needs proving on a larger scale.

## Introduction

Syphilis is a multi-stage disease caused by the spirochete *Treponema pallidum* subspecies *pallidum* (*T. pallidum*) which is transmitted both sexually and from mother-to-child [1]. From the late 1990s, the United Kingdom recorded large (ten-fold or more) increases in diagnosed cases during epidemics characterised by male-to-male transmission and high rates of HIV-1 co-infection (currently 64% in the UK) [1, 2]. Globally, the disease has remained prevalent with an estimated incidence of 10.6 million cases each year [3]. This resurgence, and the need to reduce mother-to-child transmission, has renewed interest in improved testing and treatment strategies. The aim of the current study was to develop a novel way of safely investigating syphilis treatments by directly measuring bacterial clearance following treatment.

The diagnosis of syphilis relies on serological tests; direct visualisation by dark ground microscopy (DGM) or PCR detection in infectious lesions [4]. Following direct contact with an infected lesion, *T. pallidum* invades at the point of entry to produce the chancre of primary syphilis and disseminates widely through the blood-stream and lymphatics, leading to the multi-system secondary stage [5]. The infection is subject to significant but incomplete immune clearance during the early stages and, if untreated, passes into an asymptomatic latent phase [6]. Monitoring treatment can be achieved clinically, by observing the resolution of symptoms and signs, and serologically by measuring a two-dilution (four-fold) fall in the titre of anti-cardiolipin tests, namely the venereal disease research laboratory (VDRL) or rapid plasma reagin (RPR) assays [7]. The requisite fall in RPR titre may not be observed for several months as seen in a recent treatment study where a fifth of the (HIV-1 uninfected) patients were termed ‘serofast’ at six months [8]. This creates uncertainty about the adequacy of treatment, especially as only a small proportion (13%) of these patients responded (serologically) to re-treatment with benzathine penicillin [9].

In recent years, PCR has increasingly replaced culture for the identification of pathogens. Quantitative PCR (qPCR) is commonly used to quantify viruses, such as HIV-1 and Hepatitis C in order to monitor treatment response. The quantification of bacteraemia by PCR not widely reported, but has been described for *Streptococcus pneumoniae, Neisseria meningitidis*, methicillin-resistant *Staphylococcus aureus* (MRSA), and *Acinetobacter baumannii* [10–13]. It was suggested for these infections that bacterial load is associated with disease severity and that the rate of clearance may predict clinical outcome. A recent meta-analysis of 46 studies found the sensitivity of *T. pallidum* PCR to be highest in primary genital or anal chancres (78.4%, 95% CI 68.2%–86.0%). In blood, the highest sensitivities found were for congenital (83.0%, CI 55.0%–95.2%) and secondary (52.2%, CI 37.3%–66.7%) disease [14]. For syphilis too, then, bacteraemia may be associated with disease activity. Quantitative measurement of *T. pallidum* in whole blood established a range of 195 to 1954 *polA gene* copies/ml in one cross-sectional study of secondary disease, and a mean of 516 *tpp047* copies/ml whole blood in another [15, 16]. Until now, serial measurements following treatment have not been made, thus a potential correlation between bacterial clearance and serologic cure was unknown.

Using qPCR, we measured *T. pallidum* bacterial load in blood and ulcer exudate samples from four patients with early syphilis both before and up to 150 hours after treatment with single-dose parenteral penicillin. We compared the rate of bacterial clearance with clinical outcome and standard serological follow-up.

## Methods

### Patients and samples

Patients with a microbiologically confirmed diagnosis of primary or secondary syphilis were invited to participate. Primary disease was defined as a genital, or peri-anal chancre in which *T. pallidum* was identified by DGM. Secondary disease was diagnosed in patients with consistent symptoms and signs in the context of a positive enzyme immuno-assay (EIA) result and a RPR titre of greater than one in eight.

Following informed consent, subjects were admitted to an in-patient facility and invited to donate 4 ml whole blood, of which 3 ml were collected into a Tempus RNA preservation tube (Life Technologies, UK), and 1 ml into EDTA. Each patient then received 2.4 megaunits of benzathine penicillin by intramuscular injection. Subsequently, blood samples were drawn, as above, two and six hours post-penicillin administration, then four-hourly for 48 hours (11 samples); six-hourly for 24 hours (four samples) and, finally, 12-hourly for up to four days (maximum eight samples). Samples were placed on dry ice immediately following collection and were processed in batches of up to eight. For the first patient, samples were collected at all time-points. Subsequently, the duration of sample collection was informed by *T. pallidum* clearance and ceased when two consecutive samples were negative by qPCR. Ulcer exudate was collected with a filter paper Snostrip. First, the ulcer was abraded with sterile gauze and saline. Next, exudate from the ulcer margin was absorbed up to the notch of the strip, the tip removed with a sterile blade and placed into 1.3ml of RNAlater solution (Ambion, UK).

Following discharge, patients were invited to attend for RPR testing at one, three and six months post-treatment. Serological cure was defined as a two-dilution (four-fold) reduction in RPR titre.

Ethical approval for the study was obtained from the NRES Committee South East Coast (Brighton and Sussex) (ref. 11/LO/0358). Informed, written consent was obtained from all study participants.

### Laboratory methods

Within 24 hours of collection, *T. pallidum* DNA was extracted from 500μl aliquots of whole blood. Aliquots were added to 500μl of lysis buffer (20mM TrisHcl, pH 8.0; 0.2M EDTA; 1%SDS) and incubated with 100μl of Proteinase K (Qiagen) for three hours at 65°C. Following extraction with a proprietary extraction method (QiAmp, Qiagen, Crawley, UK), DNA was purified by ethanol precipitation and resuspended in 60μl of elution buffer (Qiagen). RNA was extracted from blood in Tempus tubes according to the manufacturer’s protocol and resuspended in 100μl of the supplied elution solution. Positive and negative extraction controls were included with each batch. DNA and RNA from Snostrip samples were extracted in a single procedure with an ‘all-prep mini kit’ (Qiagen). Snostrips in RNAlater were centrifuged at 14,000g and 4°C for 30 minutes and the upper 1.1ml of RNA later discarded. The remaining 200μl were added to 600μl or buffer RLT (Qiagen) which contained 0.143M beta-mercaptoethanol. Following homogenization with a Qiashredder column (Qiagen) the resultant DNA/RNA mixture was separated and extracted according to the manufacturer’s protocol. RNA and DNA were eluted into 60μl and 100μl of RNAse-free water, respectively.

*T. pallidum* DNA quantification was achieved with qPCR using the CFX real-time system (Biorad) to detect amplification of the *tpp047* gene, which encodes a conserved 47 kDa outer membrane protein. Each reaction contained 12.5μl 2x Quantitect PCR mix (Qiagen); 0.4μM of the primers TP1 (5-CGAGGAATACAAGATTACGAACG-3) and TP2 (5-ACGTGCAGAAAAACTATCCTCAG) and 0.2μM of the hydrolysis probe TP_P (FAM-CGGCCTCGCTCAGAGATGAGC-TAMRA). Primer specificity had been assessed previously [15]. Cycling conditions consisted of 15 minutes at 95°C then 44 cycles of 95°C for 15 seconds and 80 seconds at 60°C. Quantification was achieved using an ‘in-run’ plasmid standard of a 1:10 dilution series from 10^5^ to ten copies per reaction of the *tpp047* target sequence. RNA quantification was performed in a single-step RT-qPCR. Each 25μl reaction contained 1μl enzyme mix (reverse transcriptase and taq polymerase) (Qiagen); 0·4mM DNTP mix; 5μl reaction buffer; 0.6μM of the primers 16S_F (CTCTTTTGGACGTAGGTCTTTGAG) and 16S_R (TCACCCTCTCAGGTCGGATA), and 0.2μM of the hydrolysis probe 16S_P3 (FAM-CGGCCTCGCTCAGAGATGAGC-TAMRA). Cycling conditions began with 50°C for 30 minutes, followed by 95°C for 15 minutes then 44 cycles of 95°C for 15 seconds and 60°C for 90 seconds. Absolute RNA quantification was achieved with a 1:10 dilution series of *T. pallidum* 16S rRNA produced using T7 polymerase on the recombinant plasmid containing the target sequence. For both DNA and RNA quantification, samples were analysed in triplicate and two no-template (water) controls were included in each experiment.

Results were analysed using SPSS v19. Statistical analysis is limited to descriptive statistics and a two-tailed student’s t-test.

## Results

### Assay performance

The *T. pallidum* qPCR was found to have an analytical sensitivity of at least ten *tpp047* copies/reaction. The mean inter and intra-assay coefficients of variation (for the detection of 10^4^ *tpp047* copies/μl) were 2.35% (std. dev. 0·63) and 0·66% (std. dev. 0·17), respectively. The quantification standard curve was 103% efficient with an average of 3.17 cycles between each 1:10 dilution. The RT-qPCR could detect a minimum of 23 16S rRNA copies/reaction with an inter-assay variation coefficient (for the detection of 2.3x10^4^ copies) of 3.05% (std. dev. 0.87) and intra-assay variation of 0.34% (std. dev 0.09). The 16S rRNA absolute quantification standard curve was 87.7% efficient with an average of 3.66 cycles between each 1:10 dilution.

To compare the kinetics of *T. pallidum* DNA and rRNA clearance, two parameters were calculated. The first, time to clearance, was the time elapsed between administration of treatment and the point half way between the first of the consecutively negative time-point and the last detectable time-point. The second, clearance half-life, was based on the exponential decay equation:

$$N(t)=N_{0}{\left(\frac{1}{2}\right)}^{t/t^{\frac{1}{2}}}$$

N(t) is the quantity remaining after a time t; N_0_ is the initial quantity, and t^1/2^ is the half-life of the decaying quantity. By measuring the coefficient of variation (R^2^) for an exponential regression line, the rate of nucleic acid clearance in both ulcer and blood samples was found to be exponential, thus half-life was an appropriate measure of clearance.

### *T. pallidum* quantification

Three of four patients recruited were found to have detectable *T. pallidum* bacteraemia pre-treatment and it was possible to document changes in the bacterial load over time. The fourth patient recruited was bacteraemic at time points four, six, eight and nine (between six and 30 hours post-treatment), but initially negative and was excluded from analyses. Baseline characteristics for all patients are presented in Table 1.

**Table 1:** Patient information: Baseline characteristics and serology.

Patient	Demographics	HIV-1	Stage/Signs	Baseline serology
**STS1**	Age 48 years; Male; White British; homosexual	HIV-1 infected ; CD4 640 cells/mm^3 ^; treated with Lopinavir/Ritonavir/Tenofovir; viral load <50 copies/ml	Secondary stage with rash affecting trunk and palms. No CNS involvement	EIA positive; TPPA positive; RPR 1:64
**STS 2**	Age 45 years; Male; White Brazilian; homosexual	HIV-1 infected ; CD4 776 cells/mm^3 ^; treated with Efavirenz/Tenofovir/ Emtricitabine; Viral load <50 copies/ml	Secondary stage with rash affecting trunk, arms and legs. No CNS involvement	EIA positive; TPPA positive; RPR 1:32
**STS 3**	Age 26 years; Male; Black British; Homosexual	Negative	Primary stage with painless penile chancre of four weeks’ duration	EIA positive; TPPA positive; RPR 1:32
**STS 4**	Age 35 years; Male; White Brazilian; Homosexual	HIV-1 infected ; CD4 820 cells/mm^3 ^; treated with Darunavir/Ritonavir/ Tenofovir/Emtricitabine; Viral load <50 copies/ml	Secondary stage with a feint rash, intermittent headache, healed penile ulcer	EIA positive; TPPA positive; RPR 1:64

**Fig 1:**
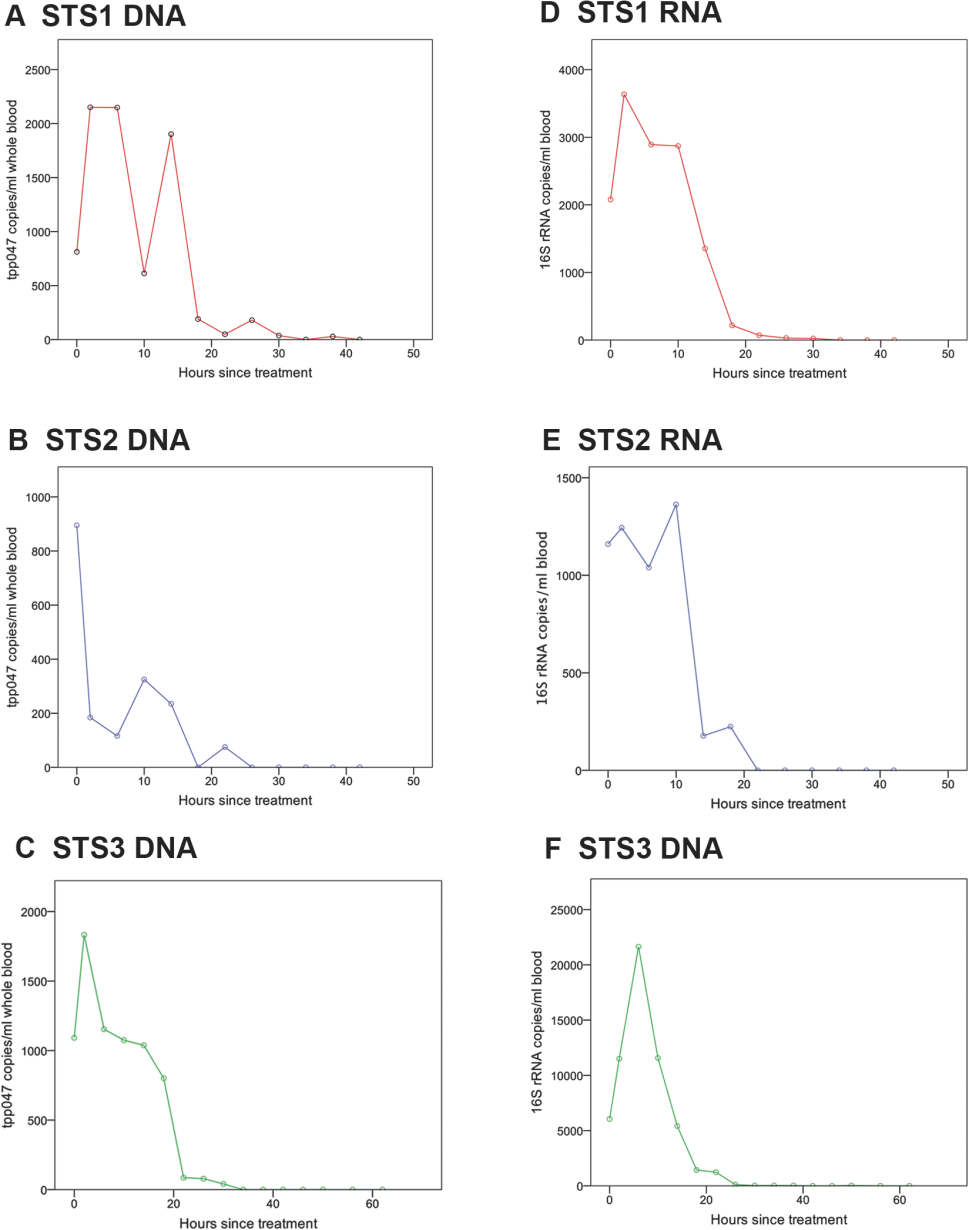
Three patients with microbiologically confirmed early syphilis were treated with a 2.4 megaunit dose of benzathine penicillin at time zero. **Panels A** to **C** represent DNA clearance for three patients and **Panels D** to **F** depict RNA clearance for the same patients. Error bars represent 95% confidence intervals.

Samples from patients STS1, STS2, and STS3 showed similar patterns of *T. pallidum* nucleic acid clearance from blood following treatment (Table 2 and Fig. 1). The mean peak *tpp047* level, which occurred two to ten hours post-treatment, was 1626 copies/ml whole blood (std. dev. 652). Peak 16S rRNA levels (8879 copies/ml whole blood, std. dev. 11109) were detected at similar time-points. While the peak of *tpp047* DNA was similar for all three patients, that of 16S rRNA for patient STS3 was ten-fold higher than for STS1 and STS2. Following peak bacteraemia, both RNA and DNA levels fell quickly, with neither detectable in any patient after 56 hours. The mean time to *tpp047* DNA clearance was 34 hours (std. dev. 8) and 29 hours (std. dev. 23.86) for 16S rRNA. The 16S rRNA time to clearance measured for patient STS3 was 56 hours, as a result of low-level (35 copies/ml) 16S rRNA detection at 56 hours following two negative samples at 42 and 46 hours. This 16S rRNA amplification at 56 hours was observed in two of three technical replicates during two separate experiments. Furthermore, both the negative Tempus™ extraction control and the two no-template PCR negative controls were negative. The mean half-life of *T. pallidum tpp047* DNA clearance from blood was determined to be 5.68 hours (std. dev 0.53) and was significantly longer than the 3.89 hours (std. dev. 0.84) calculated for 16S rRNA (p = 0.035, two-tailed t-test).

**Table 2:** Measurement of *T. pallidum* clearance post-treatment.

Patient		STS1	STS2	STS3	Mean	Std. dev.
**Blood DNA**						
	*t* ^*1/2*^ *(hours)*	5.79	6.15	5.11	5.68*	0.53
	*Peak bacteraemia (tpp047 copies/ml)*	2151	895	1832	1626	653
	*Peak Bacteraemia (hours)*	2	0	2	1.33	1.15
	*Time to clearance (hours)*	40	24	32	32.00	8.00
**Blood RNA**						
	*t* ^*1/2*^ *(hours)*	3.87	3.07	4.75	3.89*	0.84
	*Peak bacteraemia (16S rRNA copies/ml)*	3635	1363	21640	8879.33	11109.30
	*Peak bacteraemia (hours)*	2	10	6	6.00	4.00
	*Time to clearance (hours)*	32	20	53	35.00	16.70
**Ulcer DNA**						
	*t* ^*1/2*^ *(hours)*	x	x	3.19	x	x
	*Peak ulcer load (tpp047 copies/strip)*	x	x	67400	x	x
	*Time of peak load (hours)*	x	x	14	x	x
	*Time to clearance (hours)*	x	x	50	x	x
**Ulcer RNA**						
	*t* ^*1/2*^ *(hours)*	x	x	4.08	x	x
	*Peak ulcer load (16S rRNA copies/strip)*	x	x	7.08e7	x	x
	*Time of peak load (hours)*	x	x	2	x	x
	*Time to clearance (hours)*	x	x	56**	x	x

* p = 0.035 (2-sided t-test of t1/2(RNA clearance) vs t1/2(DNA clearance)

** The last sample collected was the first negative sample.

In order to exclude the possibility of a late recrudescence in bacterial load, blood from patient STS1 was sampled for both DNA and RNA quantification until 150 hours post-treatment. Neither was detectable following initial clearance at 32 (RNA) and 40 (DNA) hours.

**Fig 2:**
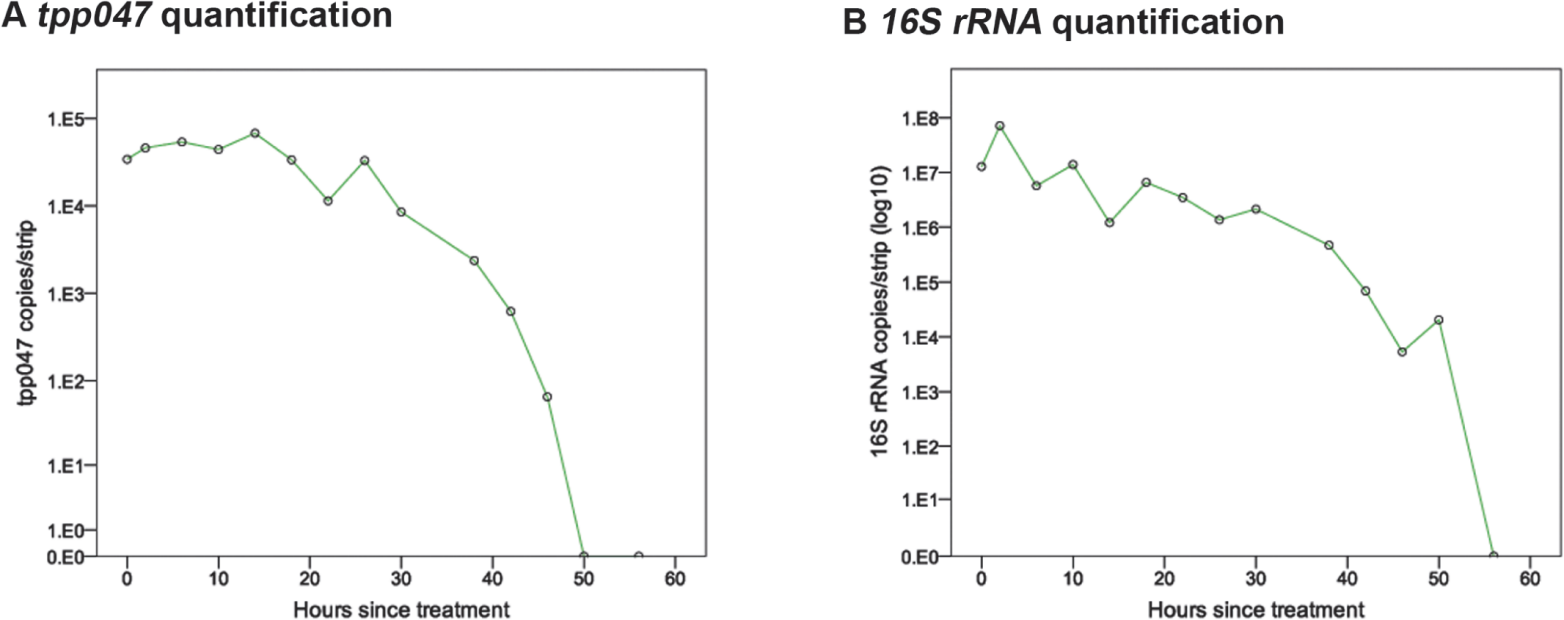
A patient presenting with a DGM positive penile ulcer (primary syphilis) was administered a 2.4 megaunit dose of benzathine penicillin at time zero. Ulcer samples for *T. pallidum* DNA and RNA quantification were collected pre-treatment and then four hourly for 56 hours. **Panel A**. presents DNA (*tpp047*) decay and **Panel B**. shows 16S rRNA decay.

Patient STS3 presented with a DGM positive genital ulcer and samples were collected concurrently with each blood sample. Table 2 and Fig. 2 demonstrate an initial ulcer *tpp047* load of 34,000 DNA copies/strip, which peaked (67,400 DNA copies/strip) at 14 hours and remained high until 26 hours, thereafter decreasing rapidly. At 50 hours following treatment, *tpp047* DNA was no longer detectable. The decay of *tpp047* DNA fitted an exponential regression line (R^2^ = 0.878) and the clearance half-life was 3.2 hours. The quantity of 16S rRNA followed a similar pattern, although this peaked earlier, at two hours post-treatment, and higher, at a level 1000 times greater than for DNA (7.08x10^7^ copies/strip). After 40 hours, the level fell exponentially (R^2^ = 0.839) to become undetectable by 56 hours. Clearance half-life was 4.1 hours.

The clearance of both DNA and 16S rRNA was measured both to improve *T. pallidum* detection (the 16S ribosomal RNA target was predicted to be present at 5000–10000 copies per organism) and to compare clearance, predicted to be shorter for RNA which is inherently less stable [17]. The half-life of *T. pallidum* DNA clearance was found to be significantly longer than 16S rRNA (5.7 hours versus 3.9 hours, p = 0·035).

### Patient progress and follow-up

Patients STS1, STS2 and STS3 all developed an inflammatory Jarisch-Herxheimer reaction (JHR) eight to 12 hours after penicillin administration. This included a worsening of rash for patients STS1 and STS2 over a one-to-two day period. At one month, clinical signs and symptoms had resolved in all three patients and serological results were consistent with cure (Table 3). This serological response was maintained at three and six months.

**Table 3:** Serological follow-up of patients

	Rapid Plasma Reagin titre			
	Baseline	One month	Three months	Six months
**Case 1**	1:64	1:4	negative	negative
**Case 2**	1:32	1:4	1:1	1:4
**Case 3**	1:32	1:2	*	negative
**Case 4**	1:64	unavailable	1:4	1:2

* Result is missing as the patient did not attend for phlebotomy

## Discussion

We present the development of a qPCR assay for quantifying *T. pallidum* in clinical samples and some early observations from a small, uncontrolled clinical study of patients with early syphilis treated with Benzathine penicillin, which highlights the potential clinical application of the assay.

Confirming adequate treatment for syphilis with serological tests can be slow (up to 12 months) and problematic due to the non-specific nature of serum RPR testing [7, 8]. Using serial sampling and novel qPCR assays, we have demonstrated clearance of *T. pallidum* nucleic acids from blood and ulcer exudate within 56 hours of treatment with benzathine penicillin in four patients. We have also described the kinetics of this response and found the half-life for blood clearance of *T. pallidum* to be 5.7 hours for DNA and 3.9 hours for RNA. For an ulcer, bacterial DNA and RNA clearance half-lives were 3.2 and 4.1 hours, respectively. All patients were cured by their treatment, showing both a clinical and serologic response.

Syphilis disease activity varies through the course of the infection giving rise to early (symptomatic) and late (asymptomatic) clinical stages. The secondary stage, characterised by systemic disease (rash, hepatitis, neurologic involvement) is arguably the most active and is the stage during which patients are most likely to be bacteraemic [14, 18]. Blood bacterial load has been measured by qPCR in a number of infections and correlated with disease activity. In patients with meningococcal disease, the level of bacteraemia at admission was significantly higher in individuals with severe disease and in those who died [11] and in MRSA bacteraemia where *mecA* DNA levels were significantly higher in non-survivors compared with survivors [12]. When the bacterial load of *Acinetobacter baumannii* in critical care patients was followed longitudinally, a slower rate of clearance was associated with increased mortality. Moreover, the use of appropriate antibiotics resulted in quicker bacterial clearance [13]. The sensitivity of *T. pallidum* PCR in blood is too low for it to be a reliable diagnostic test, especially in primary and latent disease. However, our data suggest that in selected patients with active early disease it may prove a useful marker of disease activity. Moreover, in the context of a clinical study of early syphilis treatment, qPCR may also conceivably be used to monitor bacterial clearance and quickly identify potential treatment failure.

The primary chancre of syphilis develops at the site of initial infection following bacterial division and invasion and, as such, has a high bacterial load. PCR detection of *T. pallidum* in these lesions is now established as a diagnostic tool for the disease. The chancre is also the most likely source of bacteria for onward transmission of the disease, thus the period of infectiousness following treatment is of interest. In the current study DNA and RNA from an ulcer were undetectable 50 and 56 hours following treatment, respectively. If proven in a larger clinical study, this may be of use for counseling patients regarding abstinence following syphilis treatment.

To our knowledge, there is no previous description of *T. pallidum* qPCR detection in animal or human samples to measure treatment response following therapy. The intensity of monitoring in this study enabled the observed rapid bacterial clearance to be measured accurately and serologic follow-up of patients was robust enabling a potential association between bacterial clearance and serologic cure to be identified. At present, our demonstration of bacterial clearance is limited to the study of four patients and requires confirmation on a much larger scale to become clinically useable. Moreover, whilst the clearance of bacterial DNA was measured, it is unknown whether the DNA detected was from living or dead organisms. As *T. pallidum* is non-culturable, Rabbit Infectivity Testing (RIT), where test animals are injected with clinical material in order to propagate and identify *T. pallidum*, could have been used to assess persistence following treatment [19, 20]. This approach would, however, have required the sacrifice of a large number of animals. A final limitation is that all four patients in the current study received benzathine penicillin treatment. Whilst this was deliberate, in order to define the precise moment of treatment, we acknowledge that bacterial clearance time following treatment with oral antibiotics may be different.

In recent clinical trials of syphilis treatments, enrolled patients waited for a minimum of six months following the administration of the trial drug before cure (defined as a four-fold reduction in RPR titre) was diagnosed [21, 22]. The investigation of new syphilis treatments is much needed, but to leave patients potentially untreated for six months while waiting for a serologic response is far from ideal. We hypothesise that for patients with early stage syphilis, which is PCR-positive at baseline, a clearance half-life of less than six hours or absence of bacterial nucleic acids at three days indicates bacterial clearance and is potentially a new measure of treatment success. In order to prove this, a larger observational study of syphilis treatment response is required. This proposed study would require both HIV-1 uninfected and uninfected patients with untreated primary, secondary and early latent syphilis. We propose that five sample time-points based on these preliminary data (pre-treatment, 12, 24, 56 and 72 hours post-treatment) should be used to compare bacterial clearance following treatment with both parenteral penicillin and oral antibiotics, such as doxycycline. Bacterial clearance would be correlated with serologic and clinical cure.
